# Silk Hydrogel-Mediated Delivery of Bone Morphogenetic Protein 7 Directly to Subcutaneous White Adipose Tissue Increases Browning and Energy Expenditure

**DOI:** 10.3389/fbioe.2022.884601

**Published:** 2022-05-12

**Authors:** Kristy L. Townsend, Eleanor Pritchard, Jeannine M. Coburn, Young Mi Kwon, Magdalena Blaszkiewicz, Matthew D. Lynes, David L. Kaplan, Yu-Hua Tseng

**Affiliations:** ^1^ Integrative Physiology and Metabolism, Joslin Diabetes Center, Harvard Medical School, Boston, MA, United States; ^2^ Department of Neurological Surgery, The Ohio State University, Wexner Medical Center, Columbus, OH, United States; ^3^ Department of Biomedical Engineering, Tufts University, Medford, MA, United States; ^4^ Center for Molecular Medicine, Maine Medical Center Research Institute, Scarborough, ME, United States

**Keywords:** silk hydrogel, adipose tissue, browning, thermogenesis, BMP7, intraadipose delivery

## Abstract

**Objective:** Increasing the mass and/or activity of brown adipose tissue (BAT) is one promising avenue for treating obesity and related metabolic conditions, given that BAT has a high potential for energy expenditure and is capable of improving glucose and lipid homeostasis. BAT occurs either in discrete “classical” depots, or interspersed in white adipose tissue (WAT), termed “inducible/recruitable” BAT, or ‘beige/brite’ adipocytes. We and others have demonstrated that bone morphogenetic protein 7 (BMP7) induces brown adipogenesis in committed and uncommitted progenitor cells, resulting in increased energy expenditure and reduced weight gain in mice. BMP7 is therefore a reliable growth factor to induce browning of WAT.

**Methods:** In this study, we sought to deliver BMP7 specifically to subcutaneous (sc)WAT in order to induce tissue-resident progenitor cells to differentiate into energy-expending recruitable brown adipocytes, without off-target effects like bone formation, which can occur when BMPs are in the presence of bone progenitor cells (outside of WAT). BMP7 delivery directly to WAT may also promote tissue innervation, or directly activate mitochondrial activity in brown adipocytes, as we have demonstrated previously. We utilized silk protein in the form of an injectable hydrogel carrying BMP7. Silk scaffolds are useful for *in vivo* delivery of substances due to favorable material properties, including controlled release of therapeutic proteins in an active form, biocompatibility with minimal immunogenic response, and prior FDA approval for some medical materials. For this study, the silk was engineered to meet desirable release kinetics for BMP7 in order to mimic our prior *in vitro* brown adipocyte differentiation studies. Fluorescently-labeled silk hydrogel loaded with BMP7 was directly injected into WAT through the skin and monitored by non-invasive *in vivo* whole body imaging, including in UCP1-luciferase reporter mice, thereby enabling an approach that is translatable to humans.

**Results:** Injection of the BMP7-loaded silk hydrogels into the subcutaneous WAT of mice resulted in “browning”, including the development of multilocular, uncoupling protein 1 (UCP1)-positive brown adipocytes, and an increase in whole-body energy expenditure and skin temperature. In diet-induced obese mice, BMP7-loaded silk delivery to subcutaneous WAT resulted in less weight gain, reduced circulating glucose and lower respiratory exchange ratio (RER).

**Conclusions:** In summary, BMP7 delivery via silk scaffolds directly into scWAT is a novel translational approach to increase browning and energy expenditure, and represents a potential therapeutic avenue for delivering substances directly to adipose depots in pursuit of metabolic treatments.

## Highlights

• BMP7 delivery to scWAT using protein-loaded silk scaffolds allows targeted browning without undesirable bone-formation.
• Silk scaffolds for drug delivery to scWAT can be done via direct injections through the skin with a hypodermic needle.
• Silk can be loaded with fluorescent tags to non-invasively monitor.
• BMP7 delivery to scWAT increased energy expenditure concurrent with increased tissue UCP1 expression.

## Introduction

Obesity and related metabolic conditions such as type 2 diabetes mellitus (T2DM) and cardiovascular disease (CVD) currently exist as global pandemics, and cardiometabolic disease is a leading cause of death worldwide. Obesity is a difficult condition to treat and reverse, due to the complex pathophysiology that involves both genetic predisposition and environmental factors such as diet, as well as dysfunction in numerous tissues and organs. Therapies that act on appetite centers of the brain are met with undesirable psychiatric side effects, thus, an approach that instead targets unhealthy adipose tissue itself is an appealing alternative option to not only reduce adipose mass, but prevent other metabolic comorbidities by improving adipose tissue function. Improvement of obesity can mitigate resulting co-morbidities such as T2DM, CVD and cardiometabolic diseases.

Bone morphogenetic protein 7 (BMP7) is capable of inducing a range of metabolically beneficial effects, including: 1) promotion of stem and progenitor cell commitment to the brown adipocyte lineage (Tseng et al., 2008); 2) differentiating brown preadipocytes into mature, uncoupling 1 (UCP1)-positive brown adipocytes (Tseng et al., 2008; Boon et al., 2013; Elsen et al., 2014; Okla et al., 2015)); 3) increasing mitochondrial activity and fatty acid utilization in brown adipocytes (Townsend et al., 2013); 4) increasing whole-body energy expenditure (Townsend et al., 2012; Boon et al., 2013; Saini et al., 2015; Townsend et al., 2017); 5) decreasing appetite (Townsend et al., 2012; Townsend et al., 2017); and 6) promoting neural plasticity (reviewed in (Jensen et al., 2021)). Since brown adipocytes are so metabolically healthy, with both anti-obesity and anti-diabetes functions (Townsend and Tseng, 2014) (Jensen et al., 2021), there is great interest in the potential therapeutic benefits of increasing brown adipose tissue (BAT) mass and/or activity in humans. Therefore, the utilization of BMP7 for the treatment or mitigation of obesity and its co-morbidities, such as T2DM and CVD, is of interest.

BMP7 is currently FDA-approved (marketed as OP-1 by Stryker Biotech), under the Humanitarian Device Exemption, for utilization in long bone nonunions and recalcitrant nonunions (White et al., 2007), but has not yet been used in human clinical trials as a weight-loss or anti-diabetes drug. While systemic administration of BMP7 has been shown to be effective at reversing diet- and genetically-induced obesity and diabetes in mice (Townsend et al., 2012), systemic administration in humans will be met with a number of obstacles, including the potential for bone-formation at higher doses (Luo et al., 1995) (Lavery et al., 2009), and the short half-life of BMP7 in circulation (Vukicevic et al., 1998). Therefore, direct and continuous release of BMP7 discretely in subcutaneous white adipose tissue (scWAT) should result in several beneficial effects of BMP7 treatment, as indicated by prior mouse studies (Tseng et al., 2008; Boon et al., 2011; Boon et al., 2013) (Townsend et al., 2012) (Townsend et al., 2017). These benefits include an increase in brown adipogenesis, reduction of white fat mass, activation of brown adipocyte energy expenditure, and weight loss, while avoiding the potentially harmful side effects of systemic administration such as osteogenesis in niches where bone progenitor cells are found.

Thermogenic adipose tissue consists of classical BAT depots, such as the interscapular depot in rodents and human babies, as well as inducible/recruitable cells that can be stimulated to appear in white fat depots, termed either ‘beige’ or ‘brite’, through the process of ‘browning’ (Townsend and Tseng, 2014). One of the most prominent thermogenic systems in brown and beige adipocytes is the regulated uncoupling of oxidative phosphorylation from ATP synthesis by uncoupling protein 1 (UCP1), a protein specifically expressed in the mitochondria of mammalian brown and beige adipocytes. UCP1 offers an efficient way to expend excess energy, although there also exist UCP1-independent means of heat production (Roesler and Kazak, 2020). UCP1 activity requires fatty acid fuels, which are in part released from white adipocytes through lipolysis. Browning may occur either via transdifferentiation of a mature white adipocyte to a UCP1-positive brown adipocyte, or via *de novo* adipogenesis differentiated from tissue-resident progenitors (Townsend and Tseng, 2014). Several growth factors, including BMP7, have been shown to promote the browning process, as does beta-adrenergic stimulation, such as with norepinephrine release to WAT and BAT after cold exposure (Townsend and Tseng, 2014). In humans, browning of scWAT was demonstrated by the presence of UCP1 in multilocular adipocytes, with increased presence of mitochondria and respiratory capacity after burn trauma (Sidossis et al., 2015), or after treatment with pharmacological agents (reviewed in (Herz and Kiefer, 2019)). Cold exposure or treatment with beta-adrenergic agonists in humans have been pursued as a means to increase browning, but may also lead to increased appetite, in addition to discomfort and potential cardiovascular risks (Schellen et al., 2012). Therefore, new non-sympathetic modes of increasing brown fat mass and activity are appealing options, especially if these therapies can also stimulate UCP1 activity and other metabolically healthy processes in adipose tissues.

Bioengineered silk protein delivery vehicles are an appealing candidate for mediating BMP7 delivery to WAT, as silk is a readily available natural resource (such as from silkworm production), is non-immunogenic, and is able to be engineered into microspheres, hydrogels and other drug delivery systems that protect the ‘drug’ from degradation, but are able to be regulated to control drug release kinetics ((Guziewicz et al., 2011; Hines and Kaplan, 2011; Pritchard et al., 2013a; Pritchard et al., 2013b; Guziewicz et al., 2013; Coburn et al., 2015; Lovett et al., 2015; Kluge et al., 2016; Zhang et al., 2017)). Silk-mediated delivery of BMP2 has already been optimized *in vitro* and in mouse models (Diab et al., 2012; Shi et al., 2013; Zhang et al., 2014; Koolen et al., 2016; Shen et al., 2016).

As we demonstrate here, specific delivery of BMP7 to subcutaneous (sc)WAT by silk-hydrogel delivery directly through the skin led to induction of browning of white fat. Silk-mediated, scWAT-specific, delivery of BMP7 also appears able to increase energy expenditure and reverse diet-induced adiposity, without the problem of bone formation that may occur with systemic administration, making it an appealing candidate for treatments aimed at augmenting brown fat amount and function.

## Materials and Methods

### Mouse Studies

Mice were C57BL6/J males or UCP1-Cre mated to Rosa-Luciferase males, and all were adults over 7 weeks old and housed in a standard 12/12 light/dark cycle starting at 7a.m., and were held at room temperature unless otherwise indicated. Those undergoing a high fat diet were fed 45% kcal from fat diet (part of the diet-induced obesity diet series) from Research Diets. Metabolic phenotyping included measurements in a Columbus Instruments Comprehensive Laboratory Animal Monitoring System (CLAMS) for assessment of VO2, VCO2, RER and Heat, using the Joslin Diabetes Center Metabolic Phenotyping Core, and these measurements were conducted just prior to tissue collection. DEXA scans (dual-energy X-ray absorptiometry) were used to quantify tissue composition (fat *vs*. lean), and was conducted the first day of CLAMS. An IVIS-CT small animal imager was used to image tissue levels of fluorescent or luciferase signals in live, anesthetized animals. Circulating insulin and glucose were measured by commercially available ELISAs in the Joslin Specialized Assay Core. All mouse studies were conducted similar to previously described methods (Townsend et al., 2012; Townsend et al., 2017). N = 6-8 mice were used per study, except IVIS imaging which included N = 3-4 mice per group. Separate cohorts were used for ipsilateral/contralateral measurements of changes to skin temperature and for imaging of silk injections, versus cohorts used for whole body metabolic assessments where bilateral silk injections were given directly through the skin to flank/inguinal scWAT.

### IVIS-CT

For acquisition of fluorescent and bioluminescent images an IVIS-Spectrum CT imaging system equipped with a CCD camera (Caliper Life Sciences) was used. Mice were sedated with 2% isoflurane, then fluorescent images and microCT images were captured. Silk hydrogels were labeled using DyLite800 beads and were imaged using the 735 nm excitation filter and the 800 nm emission filter. Fluorescent data was projected onto microCT imaging using Living Image software to generate 3 dimensional projections. Immediately after fluorescent imaging, D-Luciferin (Perkin Elmer) was diluted to 3 mg/100 µL in normal saline and 0.6 mg of D-Luciferin was administrated intraperitoneally. 15 min after D-Luciferin injection, bioluminsecent images were captured without an emission filter or any excitation light.

### Extraction of Silk Fibroin

Silk fibroin from B. mori silkworm cocoons was isolated as previously described (Rockwood et al., 2011). Briefly, cocoons were cut to approximately 1 cm^2^ pieces and boiled in 0.02 M Na_2_CO_3_ solution for 30, 40, 60 min to extract the silk fibroin (referred to as silk). The silk fibers were washed using deionized water (PicoPure^®^ water purification system, Hydro Service and Supplies, Durham, NC) and air dried. The dried silk fibers (5 g) were dissolved in 20 ml 9.3 M LiBr at 60°C for 3 h followed by dialysis (Pierce 3.4 kDa MWCO dialysis cassette; Fisher Scientific, Pittsburg, PA) for 2 days with at least 6 water exchanges. The resulting aqueous silk solution was stored at 4°C for future use.

### BMP7 Loaded Silk Formulations

For BMP7 loaded silk particles embedded within silk hydrogels, BMP-7 loaded silk microparticles were fabricated following a previously published poly (vinyl alcohol) ((PVA)/silk particle formulation procedures (Wang et al., 2010)). A 1% 60 min extract silk solution, 1% 40 min extract silk solution, and 1% PVA was sterile filter and handled aseptically thereafter. To prepare the particles, equal volumes of 1% 60 min extract silk solution and 1% 40 min extract silk solution were combined to a final volume of 50 ml. To this solution, 0.5 ml of 5 mg/ml sterile bovine serum albumin (BSA) was added. To 30 ml of this silk/BSA solution, 0.5 ml of 1 mg/ml BMP-7 was added; this solution was combined with 120 ml 1% PVA solution and allowed to mix for 2 h at room temperature. The solution was transferred to Petri dishes to allow for particle formation and solvent evaporation. After complete solvent evaporation, the resulting film was removed from the Petri dish using sterile forceps and transferred to a 50 ml conical tube to which 45 ml sterile deionized water was added to isolate the particles. The particle suspension was centrifuged for 10 min, the aqueous phase removed, and the particles washed two more times with ultrapure water. The resulting particles were allowed to air dry. For the pilot animal study, the particles were resuspended in sterile PBS at 15 mg/ml and centrifuged for 3 min. The particles were resuspended at 150 mg/ml. Separately, 6% 60 min extracted silk was probe sonicated, as described below, and placed on ice to form a pre-gelled silk solution. The pre-gelled silk solution was mixed 2:1 with the silk particles and maintained on ice. Ten microliters (0.5 mg silk particles) of the particle-loaded pre-gelled silk solution was injected.

To generate BMP7-loaded silk hydrogels, 2 ml of 3% or 6% silk solution was probe sonicated (Branson 450 Probe Sonifier, Branson Ultrasonics, Danbury, CT) at 30% amplitude for 30 min for two cycles to form a pre-gelled solution maintaining on ice between cycles and after sonification. BMP7 was added to the pre-gelled silk solutions at a final concentration of 120 μg/ml and maintained on ice for *in vivo* studies.

### *In vitro* BMP7 Release Studies

For silk particles loaded with BMP7 and suspended in silk hydrogel, 10 μL was transferred to low-protein bind Eppendorf tubes and allowed to gel at room temperature.

For silk hydrogels loaded with BMP7, 20 µL of the BMP7-loaded pre-gelled solution (2.4 µg BMP7) was transferred to low-protein bind Eppendorf tubes and allowed to gel at room temperature for 4 h and stored at 4°C overnight. *In vitro*, BMP7 release studies were performed by adding 1 ml PBS to each Eppendorf tube containing the BMP7-loaded hydrogels. At periodic time points, 0.950 ml PBS was removed and replaced with fresh PBS to continue the release study. BMP7 release was determined using a BMP7 ELISA kit (R&D Systems Inc., Minneapolis, MN, United States) following the manufacturers protocol.

For *in vivo* studies, 10 µL of the 3% silk formulation (1.2 µg BMP7 or empty silk control) was prepared aseptically after sterile filtration of the silk solution. The pre-gelled solutions were loaded into insulin syringes. Syringes were maintained at room temperature for 2 h (time required to observe gelation of test tube sample via inversion testing) to allow for gelation and stored at 4°C.

For *in vivo* imaging, soluble silk was labeled with a DyLight 800 antibody labeling kit (ThermoFisher, Waltham, MA, United States) following the manufacturer’s protocol. The labeled silk was combined 1:100 with unlabeled silk and subsequently used for fabricating silk hydrogels.

### Adipose Histology and Immunostaining

Tissues were fixed in 10% normal buffered formalin followed by paraffin-embedding and slicing at 7uM in a microtome. Tissue sections were mounted on slides for immunostaining, including hematoxylin to assess tissue histology, UCP1 (as described in (Townsend et al., 2012; Townsend et al., 2017)) and the nuclear fluorophore DAPI.

### Statistics

Statistics were conducted as described in (Townsend et al., 2012; Townsend et al., 2017), with ANOVA (continuous data in line graphs) or student’s *t*-test with Fisher post-hoc (bar graphs) as appropriate.

## Results

### Engineered BMP7 Silk Hydrogel Delivered Directly to scWAT in Mice

In order to avoid undesirable effects of BMP7, including bone formation, we sought a method to deliver BMP7 directly to scWAT depots in order to promote brown adipogenesis. To start, we employed silk microspheres, which were loaded with BMP7 or vehicle. *In vitro* BMP7 release rate from the microspheres measured by ELISA mimicked the dose we utilized to promote brown adipogenesis from progenitor cells in culture (Townsend et al., 2013). After optimization, *in vivo* studies were employed. Aqueous silk hydrogel solution was loaded with vehicle or BMP7 microspheres and sonicated in order to start the gelation process. While silk is kept cold, gelation is slow, but when the silk reaches body temperature gelation speeds up and allows the solution to firm itself in place at the site of delivery (Supplementary Figure S1A).

Surgical incisions above the flank/inguinal scWAT depot allowed delivery of the silk microspheres to the adipose. The right contralateral scWAT depot received BMP7 and the left contralatel depot received vehicle-loaded microspheres. Silk microsphere *in vitro* release kinetics are presented in Supplementary Figure S1B to the left. Skin temperature was compared between the left and right side 30 days after silk delivery, and 5 out of 6 mice had higher flank skin temperatures on the side that received BMP7 (Supplementary Figure S1B, right panel). Histological analysis of the scWAT depots at the conclusion of the study demonstrated an increase in multilocular (Supplementary Figure S1C) and UCP1-positive (Supplementary Figure S1D) cells in the depot that received BMP7.

**FIGURE 1:**
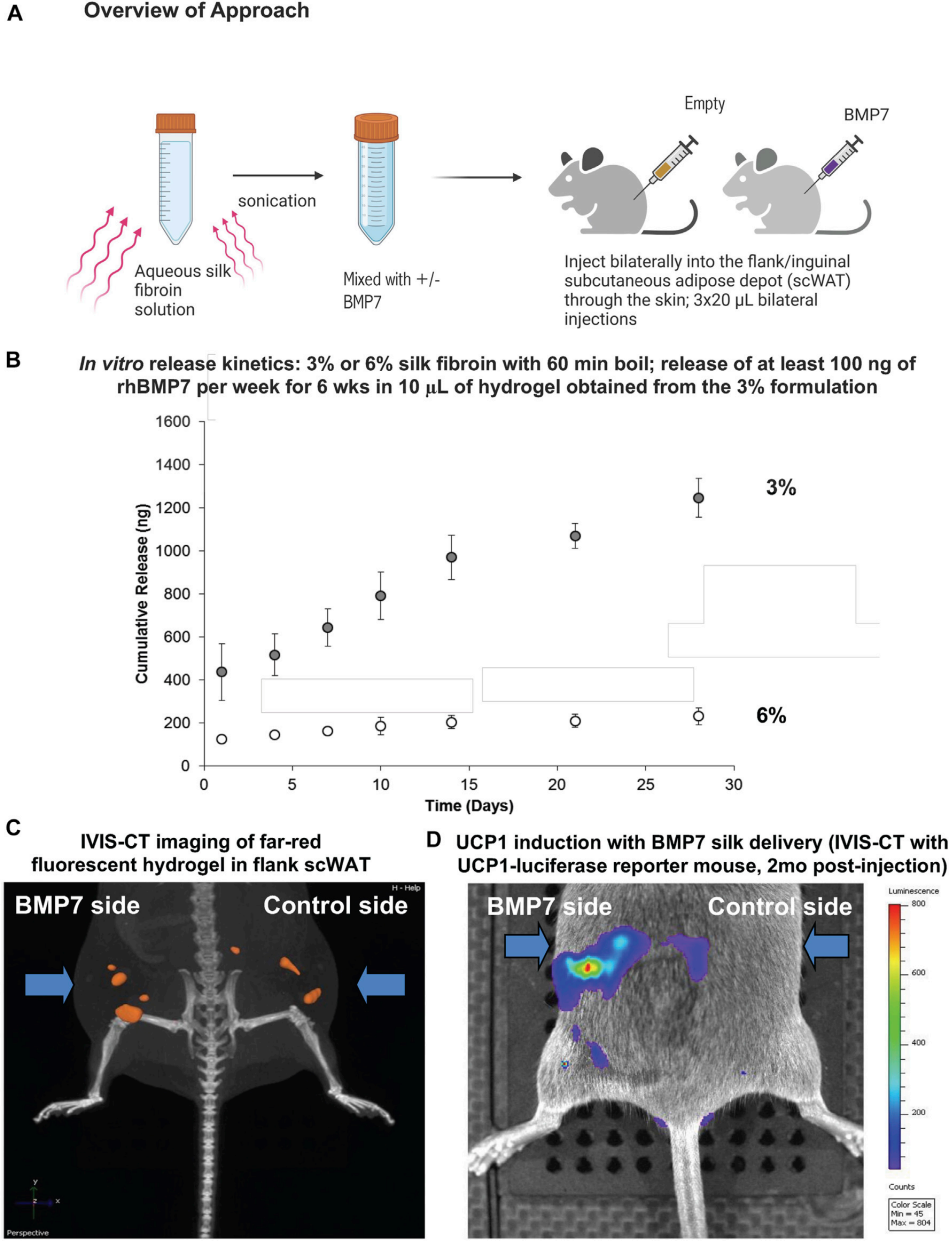
Silk hydrogels for direct delivery of BMP7 to scWAT. **(A)** Overview of the approach: aqueous silk fibroin solution is mixed with either BMP7 or vehicle in advance. On the day of the injection, the solution is sonicated in order to start the gelation process. The hydrogel remains fluid when kept on ice. Injections are performed through the skin, and we deliver three 20uL volumes of hydrogel per side, distributed anterior to posterior along the flank adipose depot. When the hydrogel reaches body temperature, it gels into place. **(B)** *In vitro* release kinetics confirmed that BMP7-loaded silk hydrogel released the target of at least 100 ng rhBMP7 per week for a period of 6 weeks when using a 10 μL hydrogel sample (ELISA data for BMP7). Data reported is for a 20 μL hydrogel. The 3% silk hydrogel formulation achieves the desired release outcomes. **(C)** IVIS-CT imaging of a UCP1-Cre/Rosa-luciferase reporter mouse, showing the far-red fluorescence of the injected silk hydrogel in the flank scWAT depots bilaterally. Representative image from N = 3 mice. **(D)** IVIS-CT imaging of a UCP1-Cre/Rosa-luciferase reporter mouse, showing UCP1 induction on the BMP7 side only (highest expression in red), 2 months post-injection. Representative image from N = 3 mice.

Since the microsphere delivery method is more labor intensive and does not allow the silk to be injected inside the scWAT depot less invasively through the skin without a silk hydrogel carrier, we moved to loading BMP7 directly into a silk hydrogel solution. This approach also allows the particles to be delivered more homogeneously to the tissue, without settling of microspheres into high-concentration areas. Similar to the microsphere experiment, the hydrogel solution is sonicated to begin the gelation process, followed by the addition of BMP7 into the pre-gelled solution, and direct injection through the skin to the flank scWAT depot (Figure 1A). Three 20 µL injections were delivered to each depot (anterior, mid, posterior in the flank depot). Hydrogel release kinetics were confirmed in preliminary studies via *in vitro* ELISA assays, with the target of releasing at least 100 ng of recombinant human BMP7 (rhBMP7) per week for a 6-week period per 10 μL hydrogel (Figure 1B). Initial tests in IVIS also confirmed that 1:100 was an optimal concentration (Supplementary Figure S2).

For initial pilot experiments, UCP1-Cre/Rosa-luficerase reporter mice were utilized (N = 3) and BMP7-loaded hydrogel was injected to the flank/inguinal scWAT, with vehicle-loaded hydrogel on the contralateral side. These hydrogels were fluorescent, which allowed us to visualize the silk placement *in vivo* using IVIS-CT intravital whole-animal imaging (Figure 1C). In addition to confirming that injections stayed in place in the scWAT depot, we were able to observe an increase in UCP1 induction via luciferase signal on the BMP7-treated side only, that persisted to 2-months post-injection (Figure 1D).

### BMP7-Loaded Silk Hydrogel Delivered to scWAT Led to Browning and Improved Metabolic Parameters in Chow-Fed Mice

**FIGURE 2:**
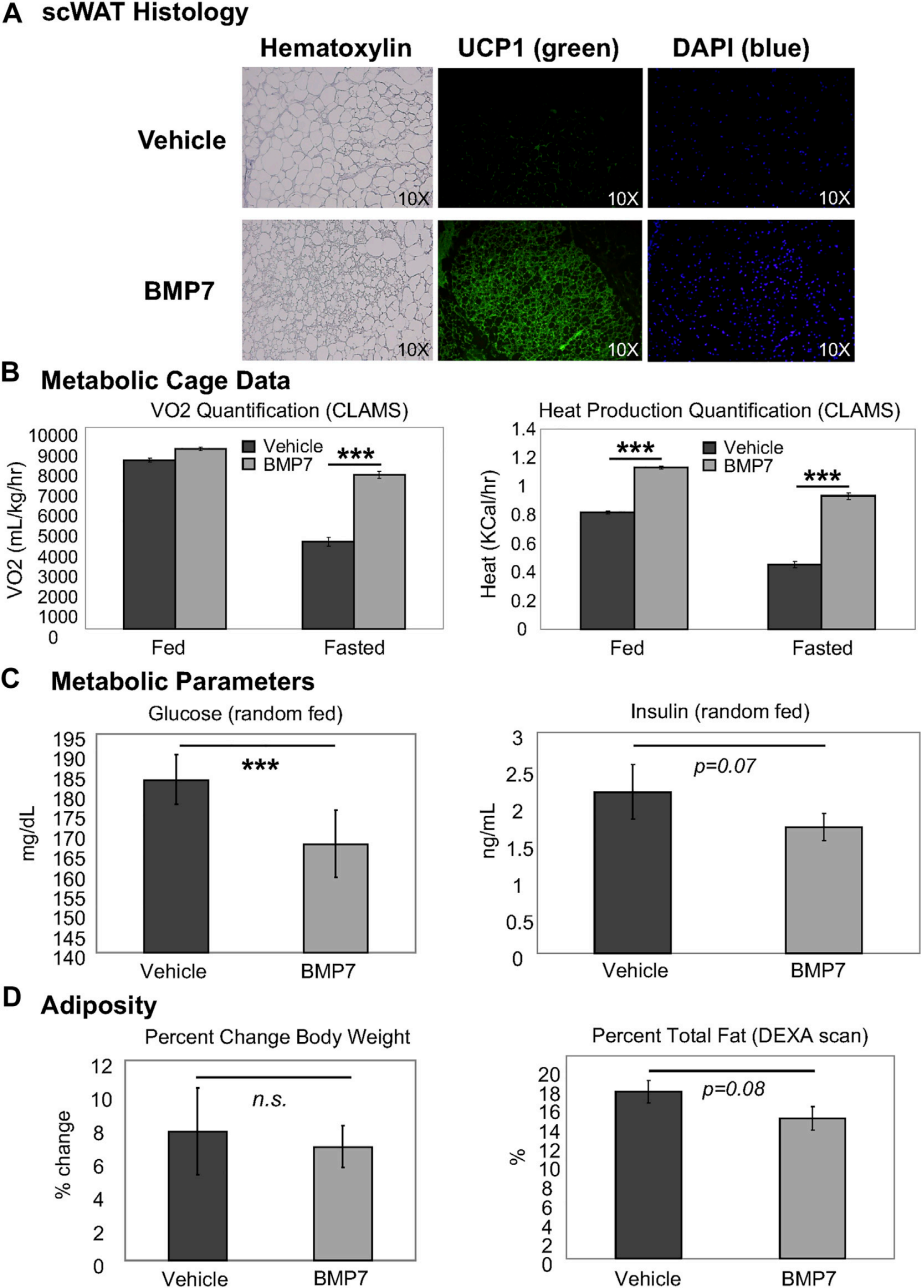
BMP7 delivery to scWAT increases UCP1+ browning and improved metabolic parameters. **(A)** Mice treated with BMP7 hydrogel to the flank scWAT depot displayed adipose histology indiciative of ‘browning’, including increased presence of multilocular cells (hematoxylin, left panels) and UCP1 positive cells (green immunostaining, middle panels). DAPI (blue, far right panels) is used to show cell nuclei. For these studies, N = 6-8 mice per group (BMP7 or empty silk hydrogel) were used. **(B)** CLAMS metabolic cages were used to measure whole body energy utilization in mice treated with vehicle or BMP7-loaded silk hydrogel to scWAT. A significant increase in VO2 was observed in BMP7-treated mice in the fasted state only, but heat was higher for BMP7-treated mice in both the fed and fasted state. **(C)** ELISA assays were used to measure circulating glucose and insulin in random-fed animals, and glucose was significantly lower in BMP7-treated animals (left panel), while insulin showed a trend to be reduced (right panel). **(D)** Across the study, chow-fed animals showed no difference in body weight with BMP7 treatment (left panel), but a trend for reduced total adiposity (body fat measured in a DEXA scan; right panel).

In chow-fed mice, BMP7 silk hydrogel treatment to scWAT led to increased multilocularity and UCP1 expression, including an increase in density of UCP1-positive cells, and increased cellularity as indicated by DAPI nuclear stain, per frame of view (Figure 2A). This indicated increased browning in the white fat with BMP7 treatment versus empty silk. Importantly, no bone formation was observed in any of the adipose depots receiving BMP7-loaded silk, and at day 20 after silk hydrogel injections circulating levels of hBMP7 measured by ELISA were undetectable (below the limit of detection for this assay’s standard curve). In separate animals during initial proof of concept studies, a subset of injections went into the leg outside of the adipose tissue, and in these animals bone formation was observed, demonstrating BMP7 bioactivity and underscoring the importance of injections directly to scWAT to avoid off-target osteogenesis. In subsequent studies with adipose delivery of BMP7, no bone formation was ever observed visually or in histological sections.

In accordance with this increase in the presence of thermogenic, energy-expending cells, the BMP7-treated mice also had higher whole body energy expenditure, as measured by VO2 (fasted state only) and heat production (both fed and fasted states; Figure 2B). Random fed glucose was reduced in BMP7-treated mice (Figure 2C) without significantly affecting insulin levels, fitting with observations that increased brown fat activity improves glucose and insulin regulation and may have anti-diabetic effects. Finally, although BMP7-treated animals did not display a reduction in body weight, they did have a trend for decreased total fat mass, as measured in a DEXA scan (Figure 2D).

### BMP7-Loaded Silk Hydrogel Improved Metabolic Health in Obese Mice Fed a High-Fat Diet

**FIGURE 3:**
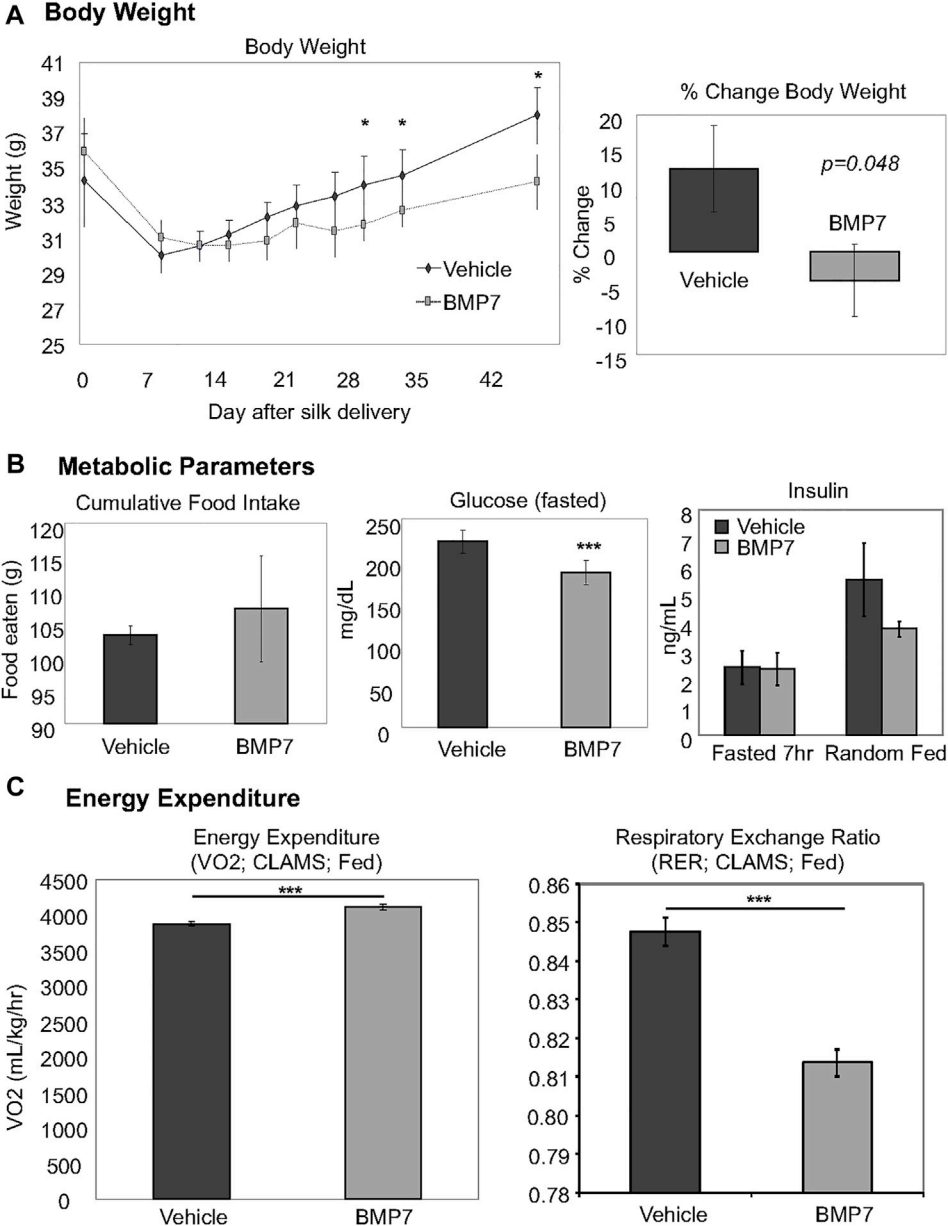
Improved metabolism in obese mice treated with BMP7 hydrogels. **(A)** BMP7-treated mice fed a 45% high-fat diet for 3 months prior to hydrogel delivery lost more weight starting at 28–45 days post-injections. For these studies, N = 6-8 mice per group were used. **(B)** BMP7-treated mice did not change food intake across the study (left panel), and showed a significant reduction in fasted blood glucose levels (middle panel) with no changes in blood insulin (far right panel). **(C)** CLAMS data indicated an increase in VO2 and a decrease in RER in mice treated with BMP7 hydrogel. A reduction in RER from 1 to closer to 0.7 indicates a greater utilization of lipids as fuel, which would be expected with higher BAT activity.

To examine whether BMP7-loaded silk hydrogel could counteract obesity and its sequelae, we fed mice with a high fat diet containing 45 kcal% fat for 3 months. BMP7 silk hydrogel delivery to scWAT at this obese timepoint led to reduced weight gain between days 28–45 of the treatment (Figure 3A, left panel, which resulted in an approximately 5% reduction in body weight across the study (Figure 3A, right panel). Early weight loss in both groups is attributed to the stress of handling and drug delivery and was not significantly different between BMP7 or vehicle treated mice. The overall weight loss due to BMP7 treatment was not due to changes in food intake (Figure 3B, left panel). Circulating glucose levels in the fasted state were significantly reduced in the BMP7-treated mice (Figure 3B, middle panel), but there was no difference in circulating insulin (Figure 3B, right panel). Energy expenditure as measured in CLAMS metabolic cages indicated increased VO2 in BMP7-treated animals in the fed state, and a reduction in respiratory exchange ration, or RER (Figure 3C). A reduction in RER from 1 to closer to 0.7 indicates a greater utilization of lipids as fuel, which would be expected with higher BAT activity.

## Discussion

Obesity and diabetes are currently global pandemics, and treatment options have thus far proven to be largely unsuccessful. Most pharmacological approaches or lifestyle modifications are met with limited success, as either side effects are high or adherence is low, respectively. Novel treatment strategies that could bypass the brain are also appealing, since many therapies targeting central appetite pathways lead to risky psychological effects. Since the rediscovery of metabolically active brown adipose tissue in adult humans, increasing the amount or activity of this thermogenic and energy-expending tissue offers an attractive way to offset the metabolic consequences of obesity and diabetes. Given its great capacity in utilizing glucose and lipids, enhanced BAT activity may be appealing for its anti-diabetic actions and improvements in metabolic phenotype, regardless of impacts on body weight and adiposity.

Given the success of silk hydrogels in delivering drugs, they also have numerous appealing characteristics for use in humans, including: eliciting minimal inflammatory response, being biocompatible and biodegradable (Li et al., 2003; Panilaitis et al., 2003; Meinel et al., 2005; Wang et al., 2008; Brown et al., 2015), representing a readily available natural resource, and existing as an FDA-approved biomaterial. Previously, silk microspheres have been used for encapsulation of BMP2 (Li et al., 2006), which remained bioactive after release and was able to induce osteogenesis of precursor cells. (BMP2 does not promote browning, but is a similar protein to BMP7.) In this study, we demonstrated that BMP7 delivery to scWAT of mice using a silk hydrogel provided an efficient means to promote browning and an improved metabolic phenotype. BMP7 has been FDA approved under the Humanitarian Device Exemption for spinal fusion surgeries, and given its numerous beneficial effects on metabolism, BMP7 is a promising candidate for human browning induction. In fact, in all of our studies, including pilot studies leading up to these published data, we did not observe any bone formation in WAT after BMP7-delivery, and we consistenly observed browning and improvement in metabolic parameters.

The duration of BMP7s effects is currently unclear, but in UCP1-reporter mice we observed UCP1 induction on the BMP7-treated side 2 months later, and high-fat fed mice displayed weight loss at 1.5 months post-treatment. Clinical studies are required to determine the optimal dosage and duration in order to sustain weight loss effects and continued improvements in metabolism. Nevertheless, the proof-of-concept studies reported here pave the way to develop effective approaches for the treatment of obesity-related metabolic diseases. It may be that humans need to receive numerous injections over time in order to sustain weight loss effects and continue to promote browning. Or, localized delivery of BMP7 may promote enough remodeling (browning and other effects) of the WAT, that systemic results are achieved through tissue cross-talk or release of BATokines to the circulation. Overall, the gradual weight loss we observed in these studies may be preferential to a more significant initial decline in adiposity, which could prevent the hypothalamus from establishing a new set point and drive up appetite counter-regulatory pathways; the more gradual weight loss we observe here could be more advantageous for longer-term sustainability. Future experiments can determine the duration of the effect and optimize treatment re-delivery strategies.

Finally, given that the research literature clearly demonstrates the importance of adipose innervation and nerve activation (in particular, norepinephrine release from adipose sympathetic nerve endings) for maintenance of UCP1-mediated thermogenesis, as well as research literature support for a neural plasticity role for BMPs (Jensen et al., 2021), we can not rule out the possibility that BMP7 is promoting innervation and neural activation of brown adipocytes after driving brown adipogenesis in scWAT. This would explain the sustained increase in energy expenditure that led to weight loss, without affecting appetite, and this would represent another benefit of BMP7 treatment, but it warrants further future exploration. In addition, further studies are needed to determine if silk-mediated BMP7 delivery is sufficient to prevent, as well as reverse, diet-induced obesity and to determine whether or not there are any potential long-term side effects. Silk hydrogel injections to scWAT through the skin may also be an avenue to deliver other treatments directly to adipose tissues, potentially as combinatorial therapies that help sustain tissue energy expenditure over longer periods.

## Conclusion

In conclusion, we provide a translational approach to deliver bioactive BMP7 protein directly through the skin to scWAT as a means of promoting browning and increased thermogenic energy expenditure, with resulting improvements to metabolic health.

## Data Availability

The original contributions presented in the study are included in the article/Supplementary Material, further inquiries can be directed to the corresponding authors.

## Supplementary Material

Supplementary Figure S1
**(A)** Approach: Pilot data using silk microspheres pre-loaded with either vehicle or BMP7. Hydrogels were surgically implanted onto the flank scWAT depot, unilaterally. **(B)** In vitro release kinetics (measured by hBMP7 ELISA) for the silk microspheres is presented on the left panel. To the right, the skin surface temperature was measured using a flat Sable systems thermocouple probe, and 5 out of 6 mice displayed higher skin temperatures on the BMP7-treated side than the vehicletreated side. **(C)** On the side receiving BMP7-loaded silk microspheres, an increase in multilocular cells was observed in the scWAT (hematoxylin) versus the vehicle-treated side. **(D)** The multilocular cells observed in the BMP7-treated side were UCP1-positive (green immunostaining), indicating they are brown adipocytes.

Supplementary Figure S2
**(A)** Transillumination biofluorescent imaging of DyLight800 in silk hydrogels injected through the skin to inguinal scWAT (unilateral on mouse right side), at 3 different concentrations ranging from 1 :10,000 to 1 :1000 and 1 :100. **(B)** Fluorescent tomography imaging of DyLight800 in silk hydrogels injected in the same mice as A, overlaid onto x-ray computed tomography to localize the signal in 3 dimensions in the adipose depot, confirming appropriate placement of the injection.
