# Supplementary figures and images for: Silk Hydrogel-Mediated Delivery of Bone Morphogenetic Protein 7 Directly to Subcutaneous White Adipose Tissue Increases Browning and Energy Expenditure
Source: Front Bioeng Biotechnol. 2022 May 12;10:884601. doi: 10.3389/fbioe.2022.884601 (PMC9135469; doi:10.3389/fbioe.2022.884601)

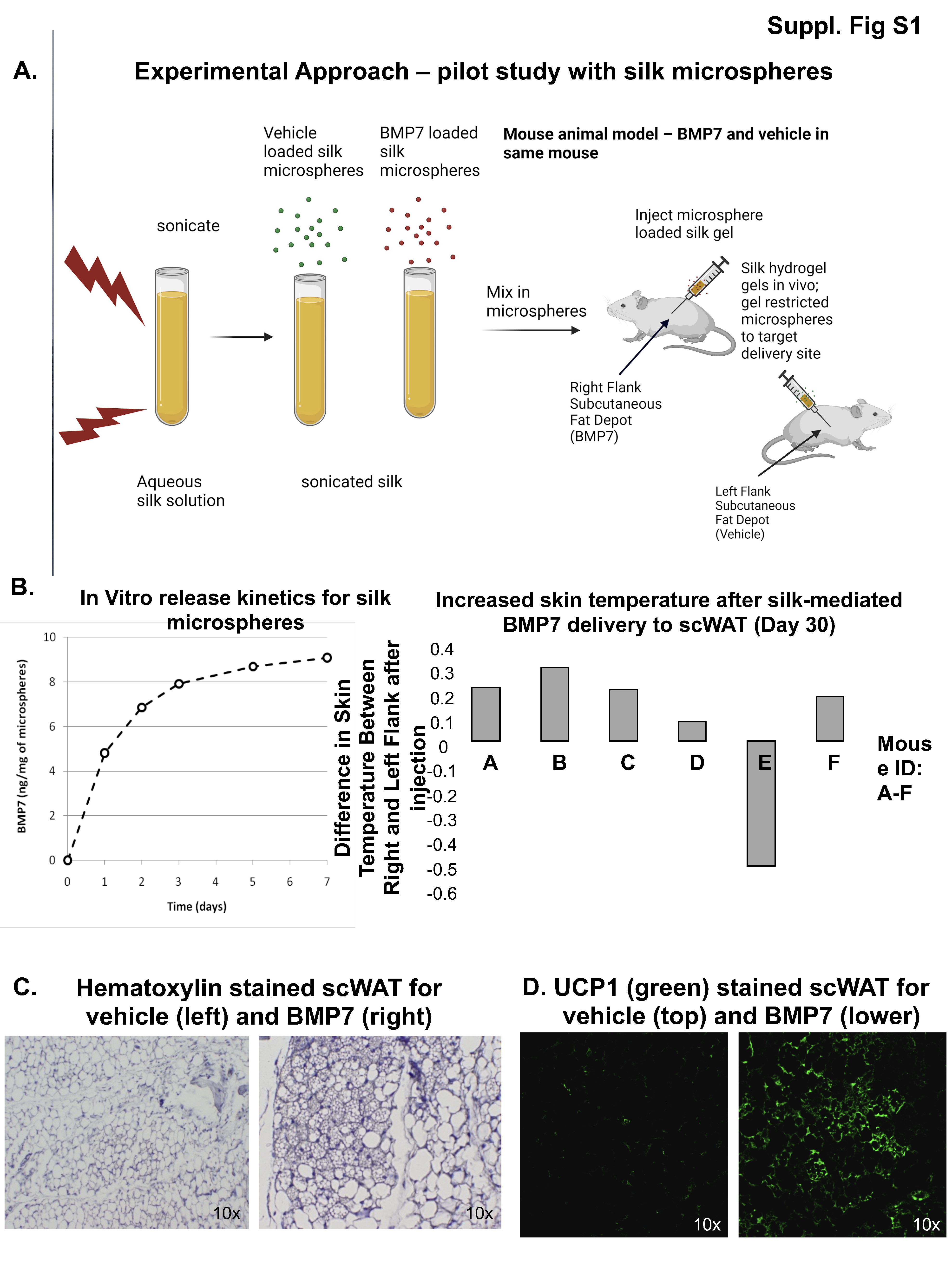

Supplement: Supplementary file 1 [file Image1.tiff]

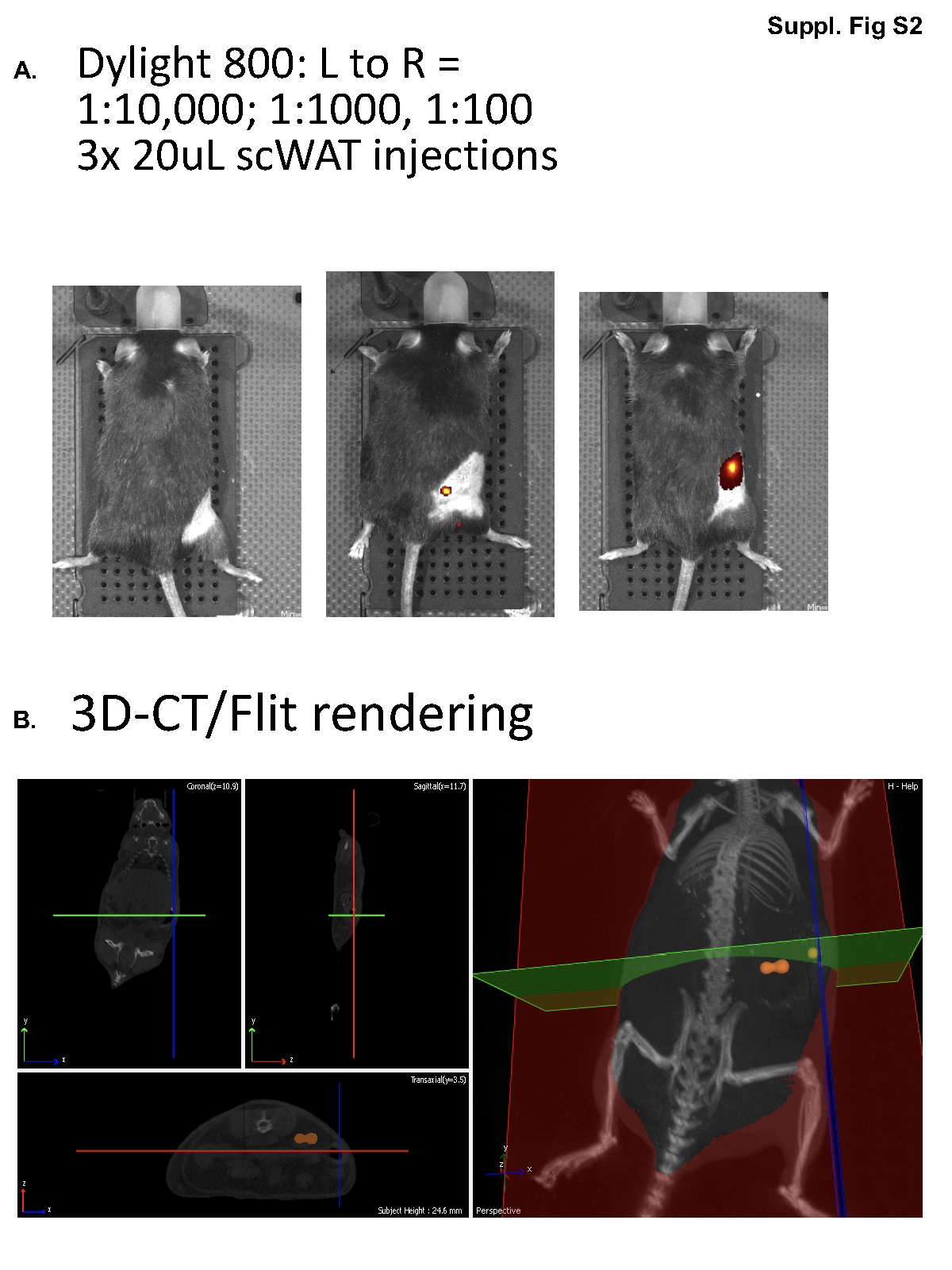

Supplement: Supplementary file 2 [file Image2.tiff]
